# Investigating the Association Between Exposure to Second Hand Smoke *in utero* and Developmental Coordination Disorder

**DOI:** 10.3389/fped.2019.00438

**Published:** 2019-11-05

**Authors:** Nadilein Mahlberg, Maeghan E. James, Rheanna Bulten, Christine Rodriguez, Matthew Kwan, John Cairney

**Affiliations:** ^1^Infant and Child Health Lab, Department of Family Medicine, McMaster University, Hamilton, ON, Canada; ^2^Faculty of Kinesiology and Physical Education, University of Toronto, Toronto, ON, Canada; ^3^Department of Kinesiology, McMaster University, Hamilton, ON, Canada

**Keywords:** developmental coordination disorder (DCD), attention deficit—hyperactivity disorder (ADHD), second hand smoke (SHS), maternal health, motor coordination

## Abstract

**Background:** Developmental coordination disorder (DCD) and attention-deficit hyperactivity disorder (ADHD) are highly comorbid in children. There is evidence linking second hand smoke (SHS) exposure *in utero* to ADHD; however, it's relation to DCD is unknown. The purpose of this study was to examine the effect of SHS exposure *in utero* in children with and without DCD.

**Methods:** This study was a cross-sectional examination of 122 children from the District School Board of Niagara (72 males, 50 females, *M*_age_ = 12.9 years) who were part of a larger, prospective cohort study. Participants were assessed for motor proficiency and intelligence and were screened for symptoms of ADHD using the Bruininks-Oseretsky Test of Motor Proficiency-Short Form, the Kaufman Brief Intelligence Inventory, 2nd edition, and the Conners' Parent Rating Scales-Revised: Short Form, respectively. Parent questionnaires were used to determine SHS exposure *in utero* as either yes or no. Multinomial logistic regression was used to examine the relationship between SHS exposure and DCD risk.

**Results:** Children exposed to SHS *in utero* were significantly more likely to be at high risk for DCD than children who were not (*OR* = 3.33, *p* = 0.004), and children exposed to SHS *in utero* were more likely to be at moderate risk for DCD in the presence (*OR* = 3.57, *p* = 0.025) or absence of ADHD (*OR* = 2.38, *p* = 0.042). However, statistical adjustment for birth weight, socioeconomic status, age, and sex reduced this effect to non-significance in the moderate risk group.

**Conclusion:** Results suggest exposure to SHS during pregnancy increases the chances of a child developing high risk DCD. While SHS exposure may increase DCD risk with and without ADHD, this effect may be explained by covariates and confounding factors. Further study is needed to examine the mechanisms linking SHS exposure *in utero* to motor coordination problems in children.

## Introduction

Developmental coordination disorder (DCD) is a neurodevelopmental disorder that affects between 5–6% of the primary school population, or approximately one child in every classroom (1). Characterized by difficulties with both fine and gross motor skills (2), children with DCD struggle with self-care tasks (e.g., dressing, using utensils), school-related tasks (e.g., handwriting, organizing seatwork, physical education class), and/or leisure activities [e.g., sports, playground activities; (3)]. An overlap between motor and attention difficulties in children has long been recognized (4) with co-occurrence rates between DCD and attention deficit hyperactivity disorder (ADHD) as high as 50% (5, 6). While the causes of DCD and ADHD are likely multifactorial, including both genetic and environmental risk factors (7), the identification of modifiable risk factors is important for prevention efforts in the future.

One such risk factor known to have a negative impact on fetal brain development is maternal tobacco smoke exposure. In Canada, it is estimated that over four million people aged 15 years and older (14.3% of the adult population) are current tobacco smokers, comprising 17.2% of men and 11.5% of women (8). Although some women cease smoking when they become pregnant, many continue to use tobacco throughout pregnancy and/or are exposed to smoking in their homes (9). Globally, more than one-third of women over the age of 15 are regularly exposed to second hand smoke [SHS; (10)]. *In utero* smoke exposure differs from inhalation exposure in that toxic substances dissolved in the blood of the mother reach the fetus through placental circulation instead of airborne exposure via the lungs. This results in carcinogenic chemicals crossing the placenta reaching the fetus at levels higher than maternal levels regardless of whether the mother is an active or passive smoker (11, 12). For example, nicotine, one of the major components in tobacco smoke, is concentrated in fetal tissue at levels as much as 15% higher than maternal levels (13). This has been shown to have adverse effects on fetal growth (14), neural development (15), and long-term cognitive and behavioral functions (16).

Maternal smoking during pregnancy has consistently been cited as a significant risk factor for ADHD, with studies reporting a two- to four-fold increased risk for ADHD in case-control, cohort, and twin studies (17–19). While recent studies suggest that the link between maternal smoking and ADHD can be attributed to unmeasured confounding familial factors (20), little research to date has examined these associations in children with DCD. Previous studies have investigated smoking in pregnancy and its impact to specific subdomains of motor function such as balance (21), coordination (22), and overall motor skills in children (23, 24). Only one study to our knowledge has examined this relationship in children with significant motor deficits (25). While results did not show a significant association between maternal smoking and DCD, the study was limited by the use of parent-reported questionnaires to classify DCD. In addition, only one study to our knowledge has examined specifically the effect of SHS *in utero* on motor development in children (26). This study found that SHS exposure *in utero* was associated with decreased gross motor skills, especially in girls, at 18 months of age. These findings warrant further investigation to examine the risk of SHS on motor skills in children.

Given that tobacco exposure during pregnancy has been linked to a range of neuro-developmental outcomes in children including ADHD, and that ADHD and DCD are highly comorbid, it is reasonable to hypothesize that SHS exposure *in utero* may also be a risk factor for DCD. Therefore, the objectives of this study were to (1) examine the effect of SHS exposure *in utero* on the risk of developing DCD, and (2) examine if there was any change in effect when comparing children at risk for DCD and typically developing children, with and without ADHD. We hypothesized that there will be a positive association between SHS exposure *in utero* and DCD, and that the association will be greater for children with comorbid DCD and ADHD. For the purposes of this study, SHS is defined as either active or passive maternal smoke exposure.

## Methods

### Participants and Study Design

The study involved a cross-sectional investigation of a subset of participants (N = 126) drawn from a larger, prospective cohort study called the Physical Health Activity Study Team (PHAST). The purpose and design of the study have been outlined in a previous publication (27). Children in the fourth grade (ages 9–10 years) were recruited from the District School Board of Niagara from 2004 to 2005. Children from 75 of 92 (83%) of schools were enrolled, resulting in a sample size of 2278 children. Data were collected at participating schools by trained research assistants over a 5-year period. The study was conducted in two phases: Phase I began in September 2004 with bi-annual, school-based health assessments in the fall and spring for all consenting children. Children were selected for Phase II using their Bruininks-Oseretsky Test of Motor Proficiency-Short Form (BOTMP-SF) scores. A total of 67 students from Phase I scoring below the 10th percentile on the BOTMP-SF in Phase I agreed to participate in Phase II. This group was designated as being at risk for DCD (DCDr) cases. Another 67 typically developing (TD) children were subsequently matched to DCDr cases of the same sex, school region^1^, and age within 6 months.

Children participating in Phase II were administered the Kaufman Brief Intelligence Inventory, 2nd edition (KBIT-2) and Movement Assessment Battery for Children 2nd edition (MABC-2) by a registered pediatric occupational therapist who was blind to the children's BOTMP-SF scores. Both the child and parent were asked to complete a series of demographics questionnaires during their appointment. After assessing motor coordination using the MABC-2, 14 children with DCDr were re-classified as TD (scoring above the 16th percentile), and 11 TD children were re-classified as DCDr (scoring at or below the 16th percentile). An additional case was excluded because of a pre-existing neurological condition. Therefore, the final sample at the end of the first year of Phase II included 63 case-control pairs. Ethics board approval was obtained from Brock University and the Niagara District School Board. Written consent was obtained from all participating parents and verbal assent was obtained from all participating children.

### Experimental Measures

#### Motor Coordination

Motor coordination was assessed using the MABC-2. The MABC-2 is a standardized test that is used to identify motor impairment in children aged 3–16 years (28) and has been identified as the criterion standard for assessing DCD in children (29). The third age band of the MABC-2 (11–16 years) was used for this study. Based on MABC-2 scores, participants were classified into one of three groups: (1) at *high risk* for DCD (high DCDr; ≤ 5th percentile; *n* = 45), (2) at *moderate risk* for DCD (moderate DCDr; between the 6th and 16th percentile; *n* = 14), and (3) TD children (>16th percentile; *n* = 63). We use the term DCDr instead of DCD because children were not assessed for full diagnostic criteria and no formal diagnoses of DCD by a physician were made. We stratified risk level based on level of motor function using established clinical cut-points from the consensus guidelines (29). It is recommended that children who score at or below the 16th percentile be closely monitored and re-assessed. Children who score below the 6th conversely are considered in the clinical range for DCD.

#### Intelligence

Intelligence was assessed using the KBIT-2, a well-recognized standardized measure of intelligence that has been used in large studies to estimate children's cognitive ability (30). The KBIT-2 measures two distinct cognitive functions (verbal and non-verbal) through three subtests (verbal knowledge, matrices, and riddles). As motor abilities may not be distinguishable from cognitive impairments (31), any children scoring below 70 were excluded.

#### Attentional Difficulties

The Conners' Parent Rating Scales-Revised: Short Form (CPRS-R:S) was selected to identify symptoms of ADHD. The short form, suitable for children 3–17 years of age, was selected in order to minimize respondent burden as it only consists of 27 items (compared to 93 items in the long form) with a completion time of 5–10 min (32). For this scale, parents were asked to rate how much each of the 27 symptoms had been a problem for their child during the last month using a 4-point scale ranging from 0 (Not True at All) to 3 (Very Much True). Each item falls into one or more of four subscales: Oppositional, Hyperactivity, Cognitive Problems, and an ADHD Index.

#### Medical History and Demographic Household Survey

A parent or guardian of the child (mother preferred) was asked to complete a questionnaire while their child was being assessed in the lab. This questionnaire was comprised of questions regarding the medical and academic history of the child and their family such as household income, child gestational age (to determine prematurity), and child birth weight, as these are identified risk factors for motor coordination problems (33, 34). To determine SHS exposure, parents were asked to indicate if the mother was exposed to “second hand smoke” on a regular basis during pregnancy (yes or no).

### Statistical Analysis

Descriptive statistics for baseline characteristics were conducted for the entire sample. ANOVA with Bonferroni *post-hoc* contrasts and chi-square analyses were used to test for group differences between high DCDr, moderate DCDr, and TD groups by age, sex, birth weight, premature birth, household income, and SHS exposure. Multinomial logistic regression was performed to examine the effect of SHS exposure *in utero* on the high DCDr and moderate DCDr groups. Covariates (age and sex) and confounding variables (birth weight, premature birth and household income) were also included in the models. Subsequently, children within the high and moderate DCDr groups were combined to form one group (moderate-high DCDr) and children in this combined group were further classified on the basis of parent-reported ADHD symptoms (moderate-high DCDr with ADHD (≥66 t-score), moderate-high DCDr without ADHD (<66 t-score), and TD). Group differences were again evaluated using ANOVA with Bonferroni *post-hoc* contrasts and chi-square analyses. Multinomial logistic regression was performed again to determine the effect of SHS exposure *in utero* on the occurrence of moderate-high DCDr without ADHD and moderate-high DCDr with ADHD, adjusting for age, sex, birth weight, premature birth, and household income. All statistical analyses were performed using SPSS version 22 (IBM Corp. Released 2013. IBM SPSS Statistics for Windows, Version 20.0. Armonk, NY: IBM Corp).

## Results

### Participant Characteristics

Of the original sample of 126 children who participated in phase II, four scored below 70 on the KBIT-2. Based on recommendations regarding the exclusion of children whose IQ is below 70 (29), these children were removed from the sample and as a result, complete data was available for 122 participants. The baseline demographic characteristics for these remaining participants are shown in Table 1.

**Table 1:** Baseline characteristics of participants from the entire sample.

**Demographic variable**	**All participants (*n* = 122)**
**Age (years)**	
Mean (SD)	12.9 (0.51)
(Min-max)	(12–14)
**Sex % (*****n*****)**	
Male	59.0 (72)
Female	41.0 (50)
**DCD classification % (*****n*****)**	
High risk DCD ( ≤ 5th percentile)	36.9 (45)
Moderate risk DCD (6th-16th percentile)	11.5 (14)
TD	51.6 (63)
**SHS exposure % (*****n*****)**	
Yes	38.5 (47)
No	59.0 (72)
Missing	2.5 (3)

*DCD, developmental coordination disorder; SHS, second hand smoke*.

A total of 38.5% (*n* = 47) of the children had a mother indicate regular exposure to SHS while pregnant. There were no significant differences in demographic characteristics among groups (high DCDr, moderate DCDr, and TD) in terms of age, sex, birth weight, premature birth, and household income (see Table 2).

**Table 2:** Baseline characteristics of participants at high or moderate risk for DCD and without DCD.

**Demographic variable**	**High DCDr (≤5th %ile) *n* = 45**	**Moderate DCDr (6th−16th %ile) *n* = 14**	**TD (>16th %ile) *n* = 63**	***p***
**Age (years)**				
Mean (SD)	13.0 (0.60)	12.9 (0.48)	12.9 (0.44)	0.355
(Min-max)	(12–14)	(12–14)	(12–14)	
**Sex % (*****n*****)**				
Male	64.4 (29)	42.9 (6)	58.7 (37)	0.357
Female	35.6 (16)	57.1 (8)	41.3 (26)	
**Birth weight (kg)**				
Mean (SD)	3.48 (0.60)	3.21 (0.66)	3.46 (0.50)	0.277
(Min-max)	(1.98–4.68)	(1.87–4.08)	(2.47–4.71)	
Missing	17.8 (8)	0	4.8 (3)	
**Premature % (*****n*****)**				
Yes	8.9 (4)	14.3 (2)	6.3 (4)	0.598^1^
No	86.7 (39)	85.7 (12)	93.7 (59)	
Missing	4.4 (2)	0	0	
**Household income % (*****n*****)**				
$0–29,999	20.0 (9)	21.4 (3)	15.9 (10)	0.627^2^
$30,000–59,999	35.6 (16)	42.9 (6)	23.8 (15)	
$60,000–89,999	20.0 (9)	21.4 (3)	19.0 (12)	
$90,000–119,999	8.9 (4)	7.1 (1)	20.6 (13)	
$120,000–149,999	2.2 (1)	7.1 (1)	6.3 (4)	
$150,000 and over	8.9 (4)	0	11.1 (7)	
Missing	4.4 (2)	0	3.1 (2)	

1 *2 cells had expected count <5*.

2 *9 cells had expected count <5*.

*DCD, development coordination disorder; TD, typicially developing; %ile, percentile*.

### SHS Exposure by Motor Proficiency

Overall, we observed significant differences in SHS exposure across groups (*X*^2^ = 8.703, *df* = 2, *p* = 0.013), with a higher percentage of mothers with children classified as high DCDr reporting regularly being exposed to SHS while pregnant (53.3%) when compared to children classified as moderate DCDr (35.7%) or TD (28.6%). Results from multinomial logistic regression confirmed SHS exposure *in utero* significantly predicted children's risk of being in the high DCDr compared to TD children. The odds of a child being at high risk for DCD when exposed to SHS *in utero* were 3.33 (95% CI = 1.47–7.57) times that of a child not exposed to SHS *in utero*. The odds ratio remained unchanged in the high DCD risk group after adjusting for age, sex, birth weight, premature birth, and household income. No significant relationships were found for SHS exposure for those in the at moderate DCDr group. Full multinomial logistic regression results are shown in Table 3.

**Table 3:** The association between SHS exposure *in utero* and DCD (at high and moderate risk) by multinomial logistic regression analysis, adjusted for age, sex, birth weight, premature birth, and household income.

	**High DCDr (≤5th %ile)**				**Moderate DCDr (6-16th %ile)**			
	**Model 1**		**Model 2**		**Model 1**		**Model 2**	
	***OR* (95% CI)**	***p***	***OR* (95% CI)**	***p***	***OR* (95% CI)**	***p***	***OR* (95% CI)**	***p***
SHS exposure	3.33 (1.47–7.57)	0.004	3.37 (1.30–8.75)	0.012	1.39 (0.41–4.72)	0.598	0.73 (0.17–3.08)	0.671
Age			1.67 (0.64–4.39)	0.299			1.04 (0.29–3.76)	0.958
Sex			0.89 (0.35–2.23)	0.802			1.57 (0.44–5.59)	0.485
Birth weight			1.52 (0.63–3.66)	0.351			0.56 (0.17–1.89)	0.352
Premature			1.34 (0.19–9.54)	0.772			2.38 (0.27–21.32)	0.438
Household income			0.89 (0.66–1.21)	0.450			0.65 (0.38–1.11)	0.113

*DCD, development coordination disorder; SHS, second hand smoke; %ile, percentile*.

### SHS Exposure by Motor Proficiency and ADHD

Due to the relatively small number of children in both the moderate and high DCDr groups, and the small number of children with symptoms of ADHD, the two groups (high DCDr and moderate DCDr) were combined into a single DCDr group ( ≤ 16th percentile) to examine the effect of SHS on comorbid DCDr and symptoms of ADHD. By combining the single DCDr group and children with symptoms of ADHD, the following three groups were created: (1) DCDr with ADHD, (2) DCDr without ADHD, and (3) TD. One participant was excluded from the analysis because they did not have the CPRS-R:S completed. Overall, chi-square analysis showed significant differences among groups for SHS exposure (*X* ^2^ = 7.192, *df* = 2, *p* = 0.027) whereby mothers of children with DCDr with ADHD (52.6%) and without ADHD (48.7%) reported regularly being exposed to SHS while pregnant compared to those in the TD group (28.6%). This suggests that mothers of children with DCDr, whether alone or in combination with ADHD, were significantly more likely to have been regularly exposed to SHS while pregnant compared to TD children. While results from the multinomial logistic regression showed an increased risk for both DCDr with ADHD (*OR* = 3.57; 95%CI = 1.18–10.34) and DCDr without ADHD (*OR* = 2.38, 95%CI = 1.03–5.46) when a child was exposed to SHS *in utero* compared to TD children, these associations were no longer significant after adjusting for age, sex, birth weight, premature birth, and household income (see Table 4).

**Table 4:** The association between SHS exposure *in utero* and DCDr with ADHD and DCDr without ADHD by multinomial logistic regression analysis, adjusted for age, sex, birth weight, premature birth, and household income.

	**DCDr (≤16th %ile) w/ ADHD**				**DCDr (≤16th %ile) w/o ADHD**			
	**Model 1**		**Model 2**		**Model 1**		**Model 2**	
	***OR* (95% CI)**	***p***	***OR* (95% CI)**	***p***	***OR* (95% CI)**	***p***	***OR* (95% CI)**	***p***
SHS exposure	3.57 (1.18–10.34)	0.025	3.17 (0.88–11.42)	0.078	2.38 (1.03–5.46)	0.042	1.96 (0.76–5.06)	0.164
Age			1.37 (0.39–4.79)	0.626			1.47 (0.56–3.83)	0.435
Sex			1.26 (0.37–4.34)	0.717			0.95 (0.38–2.37)	0.915
Birth weight			0.61 (0.18–2.05)	0.427			1.50 (0.63–3.57)	0.363
Premature			0.56 (0.04–8.08)	0.672			2.55 (0.41–15.84)	0.315
Household income			0.79 (0.50–1.25)	0.306			0.85 (0.63–1.16)	0.305

*DCD, development coordination disorder; SHS, second hand smoke; ADHD, attention deficit hyperactivity disorder; %ile, percentile*.

## Discussion

DCD and ADHD represent two prevalent neurodevelopmental disorders that may be related through common causal pathways (35), and while prior research has already investigated the possible link between SHS exposure *in utero* and the occurrence of ADHD in children, there is limited research in the area of DCD. However, given that ADHD and DCD are highly comorbid, it is reasonable to presume that SHS exposure *in utero* could also be a risk factor for DCD. Findings of the current study confirm that regular SHS exposure *in utero* is associated with an increased risk for moderate to high DCDr, with or without the presence of comorbid ADHD. Overall, these findings suggest that while active smoke exposure is important, passive smoke exposure should also be considered, as evidence shows that many of the carcinogens and toxic chemicals in smoking are also found in SHS (36).

Results of the current study are in line with findings from the few studies that have investigated maternal smoking and exposure to SHS during pregnancy, and physical control and coordination in children (21, 22, 26, 37). While these studies considered motor proficiency as a broad continuum and were not specific to those with severe motor deficits (e.g., children scoring at or below the 6th percentile), the current study offers further evidence of the relationship between SHS exposure and DCD risk using assessments of motor skills and clinical cut-points. Results of the current study contrast findings from Larsen et al. (25), who found maternal smoke exposure was not related to DCD status; however, these discordant findings may be due to the different methods used in assessing DCD, as the current study was not reliant on self-reported motor deficits.

Interestingly, children at high risk for DCD—those scoring at or below the 6th percentile on the MABC-2—were significantly more likely to have been exposed to SHS *in utero* than children scoring between the 6th and 16th percentile, what we termed as moderate risk for DCD and TD children. This suggests that there may be a dose-response relationship between maternal SHS exposure during pregnancy and the severity of motor impairments in children with DCD, though more research is needed to confirm this possibility.

As hypothesized, when compared to TD children, SHS exposure *in utero* was found to be a significant predictor of DCDr, whether on its own or in combination with ADHD. However, this association was no longer significant for the DCDr and ADHD co-morbid group when age, sex, premature birth status, and household income were included as statistical controls. Previous research has shown that certain biological factors can have an impact on both DCD and ADHD. For example, DCD (38) and ADHD (39, 40) are both more common in boys than in girls, and children born premature are at a greater risk of both DCD and ADHD (41, 42). Therefore, it is plausible to conclude that other factors may be influencing the relationship between SHS and comorbid ADHD and DCD. The present study supports findings by Skoglund et al. (20), whereby familial factors such as household income, as well as biological factors such as gender, age, and birthweight, may be accounting for some of the heightened risk for co-morbid DCD and ADHD.

Our findings suggest that limiting or eliminating exposure to SHS should be a priority for prevention of DCD and possibly ADHD in children. While substantial progress has been made to control SHS exposure in public areas and the workplace, restricting SHS exposure in the home is much more challenging. However, only by making the home completely smoke-free can SHS exposure be eliminated (43). Our study is not only one of the first to investigate the effects of SHS exposure *in utero* on the occurrence of DCD, but also provides further supporting evidence for the need to eliminate all sources of SHS exposure, especially within the home environment.

## Limitations and Future Directions

While this study provides important and novel findings, several limitations need to be acknowledged and addressed. First, we categorized children based on levels of risk for DCD because we were unable to determine whether these children met the full criteria of the Diagnostic and Statistical Manual of Mental Disorders, 5th Edition for DCD (1). Although this is not uncommon in relation to other studies (2), future work should include assessment of the impact motor coordination problems have on normal activities of daily living. In the current study, children with other medical diagnoses were not included, a measure of IQ was obtained, and a common standardized measure of motor impairment was used to fulfill the other diagnostic criteria for DCD. Also, many research studies conducted in this population of children have used only an assessment of motor ability to identify children with DCD [e.g., (2, 44, 45)].

A second limitation of the study is that children were identified with ADHD using the CPRS-R:S, a parent-completed tool. Typically, when using the CPRS-R:S tool to diagnose ADHD, its interpretation requires the integration of information from multiple sources, such as parents, teachers, and expert physicians. As only the parent perspective was obtained in this study, our identification of children with ADHD should be considered preliminary and not a diagnosis.

A third limitation was the definition of maternal SHS exposure prenatally. Mothers were asked if they were prenatally exposed to SHS on a regular basis, where “regular” was not defined. Maternal SHS exposure was categorized as “exposed” or “not exposed,” and information on frequency, quantity, and patterns or types of exposure was not collected. This broad categorization may mask the effect of low-level exposure, and also omit important information on potential dose-response. In addition, this study relied on maternal report of smoke exposure during pregnancy, which may have resulted in underreporting due both to recall bias and social desirability bias (avoidance of possible negative reactions to the stigma related to smoking or being exposed to smoke during pregnancy). If underreporting did occur, the strength of the effect of maternal SHS exposure on DCD is likely, if anything, to be an underestimate of the true association. The survey used in the present study also did not include measures of maternal smoking; therefore, we cannot disentangle the effects of SHS from maternal smoking. Future work should include measures of both.

Notwithstanding the limitations of this study, to date, this is one of the first studies attempting to examine the effects of SHS exposure *in utero* on the occurrence of DCD risk in children. The results of this study are compelling, in that SHS exposure *in utero* appears to be a strong predictor of DCD risk. While SHS exposure during pregnancy is likely not the sole cause of DCD, it does appear to be an important contributor for this prevalent developmental disorder. While further research is required to replicate these study findings with varying study populations and to overcome the aforementioned limitations, results of the current study support the need for strategies to improve maternal health and education and specifically, the prevention of smoking or exposure to smoke during pregnancy.

## Data Availability Statement

The data are not publicly available due to ethical requirements and privacy restrictions (e.g., data contains information that could compromise research participant privacy/consent). Requests to access the datasets should be directed to the corresponding author (john.cairney@utoronto.ca).

## Ethics Statement

Ethics board approval was obtained from Brock University and the Niagara District School Board. Written consent was obtained from all participating parents and verbal assent was obtained from all participating children.

## Author Contributions

NM and JC contributed to the conception and design of the study. NM organized the database and ran all statistical analyses. NM wrote the original draft of the manuscript. MJ and RB wrote and updated sections of the original manuscript. MJ, RB, and MK made substantial revisions to the original draft. All authors contributed to manuscript revisions, read and approved the submitted version.

### Conflict of Interest

The authors declare that the research was conducted in the absence of any commercial or financial relationships that could be construed as a potential conflict of interest.

## Footnotes

^1^A few cases could not be matched to children from the same school. In these situations, controls from a school within the same school region and closest proximity were selected.
